# Recent advances in oncolytic virus combined immunotherapy in tumor treatment

**DOI:** 10.1016/j.gendis.2025.101599

**Published:** 2025-03-12

**Authors:** Xiaoli Zhou, Shunfeng Hu, Xin Wang

**Affiliations:** a Department of Hematology, Shandong Provincial Hospital Affiliated to Shandong First Medical University, Jinan, Shandong 250021, China; b Department of Hematology, Shandong Provincial Hospital, Shandong University, Jinan, Shandong 250021, China; c Taishan Scholars Program of Shandong Province, Jinan, Shandong 250021, China

**Keywords:** Clinical trials, Combined therapy, Immunotherapy, Oncolytic virus, Tumor treatment

## Abstract

Oncolytic viruses (OVs), a kind of emerging therapeutics for treating tumors, are characterized by high replication efficiency, superior killing effects, and few adverse reactions, which have shown great application prospects in preclinical tumor treatment trials. To overcome the limitations of OV monotherapy, recent studies have found that combination therapy with other anti-tumor therapeutics, especially with immunotherapy, yields promising outcomes in tumor eradication. Due to the advancements in genetic engineering, the combination of OVs with novel immunotherapy, including cellular immunotherapy, adoptive cellular immunotherapy, immune checkpoint inhibitors, cancer vaccines, cytokines, and bi- or tri-specific T cell engagers, has greatly improved clinical outcomes and quality of life of tumor patients. In this review, we systematically summarize the latest progress of OVs combined with immunotherapy in tumor treatment and highlight the future directions of the combination strategies, which will promote the clinical application of OVs in tumor therapy.

## Introduction

In 1904, a leukemia patient developed a decrease in leukocyte count after an influenza virus infection.^1^ The researchers then posed a novel hypothesis: would viruses be able to kill tumor cells specifically? The concept of oncolytic viruses (OVs) was thus proposed. During the second half of the 20th century, people began to use OVs for therapy, but due to limited technology, resulting in poor treatment effects and considerable side effects, oncolytic virus therapy (OVT) has been underappreciated. Later, with the continuous development of virology and genetic engineering technology, the effectiveness, specificity, and safety of OVs in tumor therapy have been greatly improved,^2^ which play an increasingly important role in immunotherapy.

OVs represent a novel class of therapeutic agents that selectively infect and kill tumor cells while sparing normal tissues.^3^^,^^4^ This selective advantage is primarily attributed to the unique biological characteristics of tumor cells, which often exhibit altered signaling pathways and immune evasion mechanisms.^5^ OVs are classified based on their genetic material: DNA and RNA (Table 1).^6^ Adenovirus, herpes simplex virus-1 (HSV-1), reovirus, and poxviruses were the most frequently used viruses in cancer clinical trials.^7^

**Table 1:** The characteristics of different kinds of oncolytic viruses.

Virus types	Virus	Examples	Characteristics	Cell entry mechanism
DNA virus	Adenovirus	DNX-2401, enadenotucirev	Intermediate-sized viruses (∼30–40 kb) with an icosahedral non-enveloped capsid characterized by three structural components, including hexon, penton, and protruding trimeric fibers responsible for their tropism.	Endocytosis
	Herpesvirus	HF 10, T-VEC	Large enveloped viruses (∼150 kb) with icosahedral cores surrounding genomes.	Endocytosis, penetration
	Parvovirus	H-1PV	Small viruses (∼5 kb) with two transcriptional units encoding non-structural and structural components of the virus.	Endocytosis
	Vaccinia	Myxoma, GL-ONC1	Large viruses (∼200 kb) that replicate in the cytoplasm; large brick-like structures of up to 450 nm in length with complex cores with either single or double envelopes.	Membrane penetration and fusion
RNA virus	Coxsackievirus	CAVATAK, PVS-RIPO	∼7.5 kb genomes that are infectious as nucleic acid surrounded by an icosahedral lattice; tropism can be defined both by viral receptors as well as the transcriptional factors required for recognition and translations of the viral internal ribosome entry site.	Micropinocytosis via epithelial tight junctions
	Measles virus	MV-NIS, PV701	Enveloped viruses (∼16 kb) that contain two envelope glycoproteins, which mediate binding and membrane fusion.	Membrane fusion
	Poliovirus	M1, Sindbis AR339, Semliki Forest virus	Spherical particles of ∼70 nM consisting of an icosahedral nucleocapsid housing an 11.5 kb genome; alphavirus nucleocapsids are surrounded by a host-derived lipid bilayer displaying heterodimeric viral envelope glycoprotein spikes.	Receptor-mediated endocytosis
	Reovirus	Pelareorep	Morphologies of remarkable complexity, containing two concentric icosahedral capsids surrounding ten linear dsRNA segments.	Endocytosis, pH-dependent fusion activation
	Rhabdoviruses	VSV-IFNβ, MG1-MAGEA3	Enveloped, bullet-shaped or rod-shaped small viruses (∼11 kb) with substantial genome plasticity.	Endocytosis, pH-independent direct fusion

**Figure 1:**
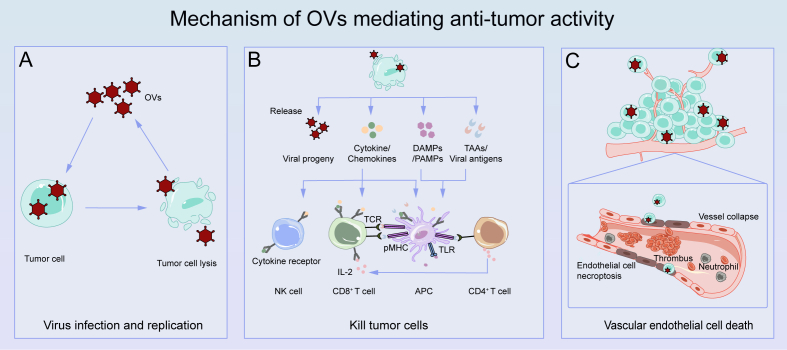
Mechanisms of OVs mediating anti-tumor activity. **(A)** OVs specifically infect and replicate in tumor cells and kill them directly. **(B)** Tumor cells infected with OVs undergo lysis and subsequent demise, thereby releasing tumor-associated antigens (TAAs), damage-associated molecular pattern molecules (DAMPs) and pathogen-associated molecular pattern molecules (PAMPs), chemokines, and cytokines. These immune-related molecules subsequently recruit APCs, NK cells, and T cells, trigger the activation of APCs, and augment the infiltration of T cells. **(C)** By infecting the endothelium of the tumor vasculature, OVs induce neutrophil influx, clot formation, and vascular collapse, resulting in tumor cell death. OVs, oncolytic viruses; TAAs, tumor-associated antigens; DAMPs, damage-associated molecular pattern molecules; PAMPs, pathogen-associated molecular pattern molecules; APCs, antigen-presenting cells; NK, natural killer.

The biology of the anti-tumor effects of OVs can be attributed to three main mechanisms (Fig. 1).^8^^,^^9^ i) Cleaving tumor cells: OVs infect and replicate within tumor cells, thus directly destroying tumor cells and releasing their progeny viruses, which further infect neighboring tumor cells.^10^ ii) Inducing systemic anti-tumor immunity^11^: The immune system receives immune “danger” signals after OVs infect tumor cells, and then activates multiple signal transduction pathways, including nuclear factor kappa-B (NF-κB), interferon regulatory factor (IRF), and mitogen-activated protein kinase (MAPK) pathways. This activation results in the extensive release of multiple molecules, including cytokines, pathogen-associated molecular pattern molecules, damage-associated molecular pattern molecules, tumor-associated antigens, and tumor-associated neoantigens. This activation subsequently promotes the production of interferons, tumor necrosis factor-alpha (TNF-α), interleukin-12 (IL-12), IL-6, and additional chemokines. Chemokines facilitate the recruitment of neutrophils and macrophages to sites of infection, while these cytokines enhance the activity of innate immune cells, including natural killer (NK) cells and dendritic cells (DCs).^12^ In addition, OV infection can induce tumor cells to undergo immunogenic cell death, stimulate the recruitment of specific immune cells, such as CD4^+^ and CD8^+^ T cells, and then induce T cells to attack uninfected tumor cells. iii) Preventing angiogenesis in tumor: OVs administered intravenously can directly infect and destroy tumor vessels without affecting normal blood vessels, thus inhibiting tumor growth.^13^

Moreover, understanding the pharmacokinetics of OVs is crucial for optimizing their therapeutic efficacy and safety profiles. The pharmacokinetics of OVs can be influenced by various factors, including the route of administration, the presence of pre-existing immunity, and the tumor microenvironment (TME). For instance, the use of polymeric materials to coat OVs improved their pharmacokinetic profiles by reducing neutralization by antibodies and prolonging circulation time in the bloodstream.^14^ Furthermore, the immune response elicited by OVs can also affect their pharmacokinetics, as the presence of neutralizing antibodies can limit the ability of OVs to spread within the tumor.^15^ Therefore, continuing to explore the pharmacokinetic properties of OVs, as well as their interactions with the immune system, is critical to improving therapeutic efficacy and reducing potential side effects.

The treatment of tumors involves different strategies, including surgery, radiation therapy, chemotherapy, targeted therapy, and immunotherapy. However, traditional therapies like chemotherapy and radiation can affect both healthy and tumor cells, leading to significant side effects.^13^ Moreover, many tumors develop resistance to these treatments over time, which can decrease their efficacy and result in recurrence.^16^ While immunotherapy holds great promise for tumor treatment, significant barriers remain, including tumor heterogeneity, immunosuppressive TME, tumor targeting, drug resistance, and the influence of gut microbiota on treatment efficacy.^16^, ^17^, ^18^, ^19^ OVs offer a promising alternative by selectively targeting tumor cells and activating the immune system. What's more, with the development of genetic engineering, OVs can be modified using gene-editing technology to improve tumor targeting and enhance anti-tumor efficacy, thus overcoming the limitations of other therapies.^20^ This modification can involve equipping OVs with transgenes, cytokines, chemokines, and bi- or tri-specific T cell engager (BiTE or TriTE) molecules.^21^ Among them, the most common transgene was granulocyte-macrophage colony-stimulating factor (GM-CSF), followed by LacZ. In conclusion, for tumor patients who have been unsuccessful with traditional therapies, OVT offers novel and promising treatment options.

Four OVs have been approved globally to treat tumors because of clinical trials that have shown positive results.^22^, ^23^, ^24^, ^25^ Although OVs have made significant progress in tumor treatment, there are still great challenges in promoting their clinical applications. Single agents have limited efficacy due to poor OV diffusion and penetration, low antiviral efficacy, delivery platforms, and patient selection.^26^ Therefore, through the integration of various strategies, including the use of carrier cells,^27^ magnetic targeting of OVs,^28^ polymer coating,^29^ and genetic modification,^20^ it is possible to enhance tumor targeting and improve the anti-tumor effects of OVs, laying the foundation for more effective and safer OVT. Furthermore, researchers have combined OVT with other therapies to improve tumor treatment and patient quality of life, including radiotherapy, chemotherapy, targeted therapy, and immunotherapy.^30^^,^^31^ An increasing number of studies have indicated that the anti-tumor properties of OVs make it an excellent partner for emerging immunotherapy, which can cooperate to boost tumor-killing efficacy and have good therapeutic potential for tumor treatment. This review focuses on the latest progress of OV-combined immunotherapy for tumor treatment in the era of precision medicine and highlights the potential of combination therapy for clinical translation, which might provide new ideas and explore new schemes for the treatment of tumor patients.

### OV-combined immunotherapy

**Figure 2:**
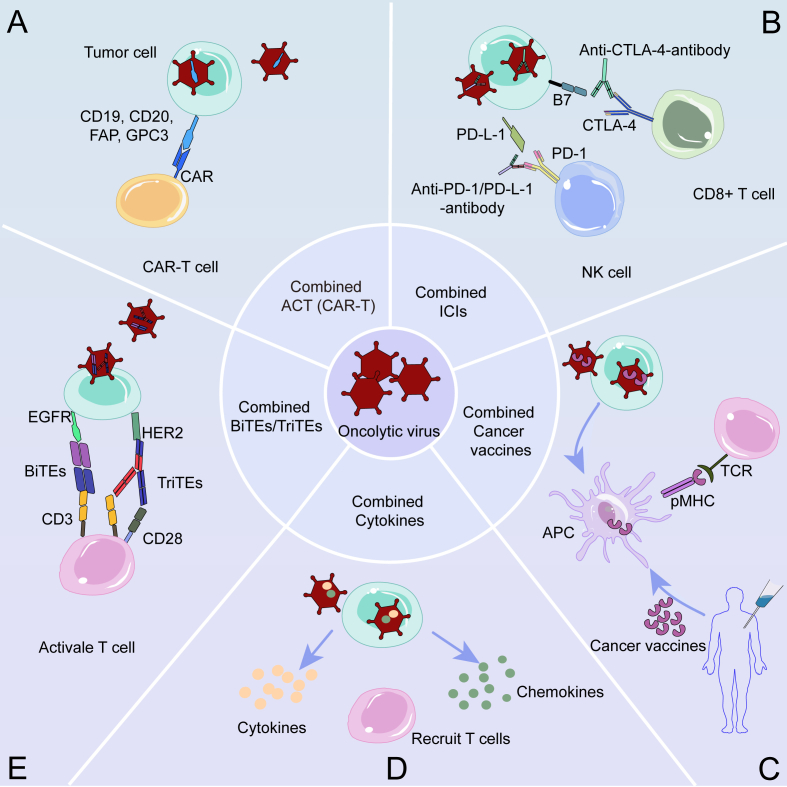
Combinations of OVs with immunotherapy for tumor treatment. **(A)** Tumor cells infected with modified OVs can express surface antigens, attracting CAR-T cells into TMEs, and enhancing their activities. **(B)** Infection with OVs increases the expression of immune checkpoints, such as PD-1/PD-L-1/CTLA-4 on tumor cells. The antibodies to PD-1/PD-L1/CTLA-4 stop the immune inhibitory signal and enhance the anti-tumor ability of OVs. **(C)** OVs in combination with cancer vaccines stimulate the immune system and destroy tumor cells. **(D)** OVs encoding cytokines can further enhance T-cell infiltration and increase anti-tumor efficacy. **(E)** BiTE or TriTE-armed OVs target the TME for immune regulation by activating T cells. OVs, oncolytic viruses; CAR, chimeric antigen receptor; PD-1, programmed death-1; PD-L1, programmed death ligand-1; TME, tumor microenvironment; CTLA-4, cytotoxic T-lymphocyte-associated protein 4; BiTE, bi-specific T cell engager; TriTE, tri-specific T cell engager.

Although OVT, as an emerging method of tumor treatment, has great potential, the efficacy of monotherapy treatment is limited at present, and it is difficult to eliminate tumors by relying only on OVT, which often needs to be combined with other therapies.^32^ The mechanisms of OVs killing tumors are different from those of other anti-tumor drugs, and the toxicity is controllable, which provides an opportunity for the combination of OVs with other therapies. According to recent reports, OVs have shown increased effectiveness when used in conjunction with other methods of treating tumors,^33^ especially immunotherapy.^34^, ^35^, ^36^ OV-combined immunotherapy approaches will be briefly discussed in this section (Table 2 and Fig. 2).

**Table 2:** Clinical trials of combination immunotherapy with OVs for tumor treatment (2022–2024).

Oncolytic virus	Immunotherapy types	Tumor types	NCT number	Clinical phase	Status	Therapeutic purpose and outcomes
onCARlytics (CF33-CD19)	CAR-T cells and blinatumomab	Solid tumors	NCT06063317	Phase I	Recruiting	To evaluate the safety and tolerability of CF33-CD19 plus blinatumomab
RT-01	ICIs (nivolumab)	Advanced solid tumor	NCT05228119	Phase I	Unknown status	To evaluate the safety and efficacy of RT-01 plus nivolumab
Oncolytic virus	ICIs (anti-PD1 antibody)	Pancreatic cancer	NCT06346808	Phase I	Not yet recruiting	To evaluate the safety and efficacy of OV plus anti-PD1 antibody and chemotherapy as preoperative therapy
H101	ICIs (camrelizumab)	Pancreatic cancer	NCT06196671	Phase II	Not yet recruiting	To evaluate the efficacy of OV plus anti-PD1 antibody
H101	ICIs (programmed death receptor-1 inhibitor)	Malignant pleural mesothelioma	NCT06031636	Phase I	Recruiting	To evaluate the safety and efficacy of H101 plus anti-PD1 antibody
CF33-hNIS	ICIs (pembrolizumab)	Gastric cancer peritoneal metastases	NCT05346484^99^	Phase I	Recruiting	To evaluate the safety and tolerability of CF33-hNIS plus pembrolizumab. Results found combination therapy significantly prolongs the survival of mice bearing gastric cancer.
ONCOS-102	ICIs (balstilimab)	Melanoma	NCT05561491	Phase II	Withdrawn	To evaluate the safety and efficacy of ONCOS-102 alone or plus balstilimab
RP2 and RP3	ICIs (atezolizumab)	Refractory metastatic colorectal cancer	NCT05733611	Phase II	Active, not recruiting	To evaluate therapy with RP2 or RP3 plus atezolizumab and bevacizumab
H101	ICIs (camrelizumab)	Recurrent cervical cancer	NCT05234905	Phase II	Not yet recruiting	To evaluate the objective response rate of H101 plus camrelizumab
TILT-123	ICIs (avelumab)	Advanced solid tumors (melanoma and squamous cell carcinoma of head and neck)	NCT05222932^91^	Phase I	Recruiting	To evaluate the safety of TILT-123 plus avelumab. Preliminary results found TILT-123 was safe and able to produce anti-tumor effects in melanoma.
RP3	ICIs (atezolizumab)	Advanced unresectable or metastatic hepatocellular carcinoma	NCT05733598	Phase II	Not yet recruiting	To evaluate the anti-tumor efficacy of RP3 plus atezolizumab
TILT-123	ICIs (pembrolizumab)	Platinum-resistant or -refractory ovarian cancer	NCT05271318^91^	Phase I	Recruiting	To evaluate the safety of TILT-123 plus avelumab. Preliminary results found TILT-123 was safe and able to produce anti-tumor effects in ovarian cancer.
RP1	ICIs (atezolizumab)	Triple-negative breast neoplasms	NCT06067061	Phase I/II	Recruiting	To evaluate the efficacy of RP1 plus atezolizumab
MEM-288	ICIs (nivolumab)	Non-small cell lung cancer	NCT05076760	Phase I	Recruiting	To evaluate the maximum tolerated dose and recommended phase II dose of MEM-288 alone or plus nivolumab
GM103	ICIs (pembrolizumab)	Solid tumors	NCT06265025	Phase I/II	Recruiting	To evaluate the safety, tolerability, and preliminary anti-tumor efficacy of GM103 alone or plus pembrolizumab
JNJ-87704916	ICIs (cetrelimab)	Advanced solid tumors	NCT06311578	Phase I	Recruiting	To determine the safety, feasibility, recommended dose, and regimen of JNJ-87704916 alone or plus cetrelimab
RP3	ICIs (nivolumab)	Locoregionally advanced or recurrent squamous cell carcinoma of the head and neck	NCT05743270	Phase II	Withdrawn	To evaluate the anti-tumor efficacy of OV plus nivolumab
BioTTT001	ICIs (toripalimab)	Peritoneal metastases from gastric cancer	NCT06283121	Phase II	Not yet recruiting	To evaluate the efficacy of BioTTT001 plus SOX and toraplizumab
T3011	ICIs (toraplizumab)	Liver metastases from colorectal cancer	NCT06283303	Phase I	Not yet recruiting	To evaluate the safety and efficacy of T3011 alone or plus toripalimab and regorafenib
Adenovirus	ICIs (anti-PD1 antibody)	Advanced malignant melanoma	NCT05928962	Phase I	Enrolling by invitation	To evaluate the safety and efficacy of adenovirus plus anti-PD1 antibody
BioTTT001	ICIs (toripalimab)	Liver metastases from colorectal cancer	NCT06283134	Phase I	Not yet recruiting	To evaluate the safety and efficacy of BioTTT001 plus toripalimab and regorafenib
OH2	Cytokines (GM-CSF)	Advanced bladder carcinoma	NCT05248789	Phase II	Recruiting	To evaluate the safety and efficacy of OH2 armed with GM-CSF
OH2	Cytokines (GM-CSF)	Non-muscle-invasive bladder cancer	NCT05232136	Phase I/II	Recruiting	To evaluate the safety and efficacy of OH2 armed with GM-CSF
Ad-TD-nsIL12	Cytokines (IL-21)	Progressive pediatric diffuse intrinsic pontine glioma	NCT05717699	Phase I	Recruiting	To observe the safety, tolerability, and toxicity of OV armed with IL-21
Ad-TD-nsIL12	Cytokines (IL-21)	Primary pediatric diffuse intrinsic pontine glioma	NCT05717712	Phase I	Recruiting	To observe the safety, tolerability, and toxicity of OV armed with IL-21
hV01	Cytokines (IL-21)	Advanced solid tumor	NCT05914376	Phase I	Recruiting	To evaluate the safety, tolerance, pharmacokinetics, and biological properties of OV armed with IL-21
RGV004	BiTEs	Relapsed or refractory B-cell lymphoma	NCT04887025	Phase I	Recruiting	To evaluate the maximum tolerated dose and dose-dependent toxicity of OV-armed RGV004

Note: OVs, oncolytic viruses; CAR, chimeric antigen receptor; ICIs, immune checkpoint inhibitors; GM-CSF, granulocyte-macrophage colony-stimulating factor; IL-21, interleukin 21; PD1, programmed death-1; BiTE, bi-specific T cell engager.

### OVs in combination with adoptive cellular immunotherapy

Adoptive cellular immunotherapy, a type of immunotherapy, involves the infusion of immune cells with anti-tumor activity into the patient to play an anti-tumor role.^37^^,^^38^ Various forms of adoptive cellular immunotherapy consist of chimeric antigen receptor (CAR) T cell therapy, NK cell therapy, T cell receptor (TCR) T cell therapy, and tumor-infiltrating lymphocytes (TIL) therapy. Due to the short survival of adoptive T cells and NK cells and the highly heterogeneous nature of tumors, there are still certain limitations to the application of adoptive cellular immunotherapy in tumors.^39^ Respective clinical trials combining OVs with adoptive cellular immunotherapy are ongoing in tumor patients and show promising anti-tumor efficacy.

### OVs in combination with CAR-T cell therapy

CAR-T cell therapy involves the genetic modification of T cells to express CARs, enabling them to identify and eliminate tumor cells that display specific antigens.^40^ CARs are artificial fusion proteins that contain both extracellular antigen recognition domains and intracellular signaling domains, designed to enhance the specificity of T cells and other immune cells.^41^, ^42^, ^43^ With the development of CAR-T cell therapy, the treatment of tumors has been revolutionized in recent decades.^44^^,^^45^ As opportunities and challenges always exist together, CAR-T cell therapy has shown promising clinical results in hematologic malignancies, with substantial challenges persisting in targeting solid tumors, including how to identify optimal antigens, effective transport, and persistence within immunosuppressive tumors.^46^, ^47^, ^48^, ^49^ The combination of OVs with CAR-T cell therapy can overcome the above barriers by sensitizing tumors to CAR-T cell recognition through up-regulating the expression of tumor antigen and immune-stimulatory molecules.^50^^,^^51^

It is advantageous to combine CAR-T cell therapy with OVs since OVs armed with cytokines or chemokines provide extended T-cell persistence and a greater anti-tumor response.^21^^,^^52^^,^^53^ To correct the poor infiltration of CAR-T cells, researchers engineered a chemokine, CXCL11, to arm oncolytic adenovirus. For example, in a mouse model of glioblastoma multiforme, OAd-CXCL11 had a strong anti-tumor effect by increasing the infiltration of CAR-T cells and transformed a cold immunosuppressive state into a hot immunosupportive one.^54^ OAd expressed TNF-α and IL-2, which promoted meso-CAR T cell accumulation in pancreatic cancer, enhanced T cell engraftment, and reduced resistance in mouse model.^55^ What's more, an oncolytic adenovirus (OAV) expressing the chemokine IL-12 (Ad5-ZD55-hCCL5-hIL12) combined with CAR-T cells significantly prolonged mouse survival and inhibited tumor growth by enhancing the infiltration of CAR-T cells. IL-12 mediated by Ad5-ZD55-hCCL5-hIL12 also induced the phosphorylation of Stat4 in CAR-T cells, thereby promoting an increased secretion of interferon-gamma (IFN-γ) by these cells in renal cell carcinoma.^56^ In a recent study, the systemic delivery of oncolytic herpes simplex virus (oHSV) using CAR-T cells enhanced the targeting of anti-tumor immuno-virotherapy.^57^ These results indicated that the combination of OVs with CAR-T cell therapy has sufficient potential and prospects for the treatment of tumors. However, CAR-T cell therapy has caused some serious adverse events, such as immune effector cell-associated neurotoxicity syndrome and cytokine release syndrome. Therefore, when using CAR-T cell therapy in combination with OVs, safety profiles and rational design need to be considered, including the optimization of delivery methods and the development of novel OVs.^50^

### OVs in combination with NK cell therapy

In recent years, in addition to CAR-T therapy, NK cell therapy has also gradually attracted attention, which can lyse target cells through two mechanisms: missing-self and antibody-dependent cell-mediated cytotoxicity.^58^ Meanwhile, NK cells selectively recognize and destroy tumor cells by interaction between activating receptors and inhibiting receptors, without prior sensitization.^59^^,^^60^ Although NK cell therapy has a broader spectrum of anti-tumor effects, many significant challenges still need to be addressed to successfully translate it from bench to bedside, especially how to enhance the infiltration of NK cells at the tumor site and increase the activity of NK cells.^61^^,^^62^ OVs can convert cold tumors to hot tumors, overcome the above barriers, and thereby enhance the tumor-killing capacity of NK cells.^62^^,^^63^

Multi-model studies have shown synergistic effects between NK cell therapy and OVT.^64^, ^65^, ^66^ In 2021, Varudkar et al proposed a hypothesis^67^ on the use of oncolytic parainfluenza virus 5 (P/V virus) in combination with PM21-NK cell therapy against lung cancer. The introduction of substitutions in the PIV5 P/V gene transformed the non-cytopathic wild-type virus into a P/V virus, which exhibited high cytopathicity and acted as a potent inducer of antiviral cytokines. Results of an *ex vivo* study showed that the P/V virus reduced tumor cell growth and increased the susceptibility of uninfected lung cancer cells to PM21-NK cell killing.^67^ Li et al mutated five sites of wild-type human IL-2 sequence and inserted the vIL-2 transgene under the E3 promoter to construct OAV Ad5/3-E2F-d24-vIL2. The vIL-2 virus elicited an up-regulation of granzyme genes while concurrently down-regulating genes associated with myeloid-derived suppressor cells. The Ad5/3-E2F-d24-vIL2 effectively mitigated the immunosuppressive TME in human ovarian cancer by augmenting the cytotoxic activities of NK cells and T lymphocytes.^65^ A recent study found that decorin-armed oAD up-regulated the expression and secretion of factors related to NK cell killing activity, promoted NK cell activation and infiltration, and enhanced NK therapy in colorectal cancer.^68^ In a study, an OAV was engineered to express a transgene encoding non-cleavable MICA (MHC class I chain-related polypeptide A), which enhanced NK cell-mediated cytotoxicity, increased IFN-γ and CD107a degranulation secretion, stimulated the immune response against tumor cells, and improved anti-tumor immune responses in mouse models.^69^ These results provided compelling experimental proof applicable for future advances in the clinical translation of a combination of OVs with NK cell therapy. Despite the positive results of multiple experiments using this combination therapy, the complex interplay between OVs and NK cells necessitates a rational design of such therapies. For instance, strategies need to be properly developed to enable NK cells to enter and target metastatic sites where the virus cannot be injected.^70^

### OVs in combination with TCR-T cell therapy

TCR^71^^,^^72^ is a heterodimeric protein located on the surface of T cells, which specifically recognizes antigens on the surface of T cells and mediates immune response. Compared with CARs, TCRs have some structural advantages, such as more subunits in their receptor structure, less dependence on antigens, and more costimulatory receptors, including CD3, CD4, and CD28.^73^ Although TCR-T cell therapy has shown clinical efficacy in most treated patients, there are still many challenges to be addressed, including insufficient and transient expression of TCR, tumor immune escape, and the lack of effective tumor-specific antigens to target.^74^^,^^75^ Overcoming these challenges will be key to achieving greater clinical success in the future.

Preclinical studies in animal models have found that the combination of TCR-T cell therapy with OVT could lead to the expected improvement in treatment response.^76^ Therefore, the infiltration and migration of genetically engineered T cells to tumors can be induced by combining OVs, thus enhancing the anti-tumor effect of TCR-T cells. Chen et al^77^ tested OV-T cell chimera (ONCOTECH) via intravenous delivery in several mouse tumor models. OVs were physically attached to T cells via their receptors (TCR or CAR) without affecting their function. *In vivo* and *in vitro* experiments showed that ONCOTECH reshaped the immunosuppressive TME and improved anti-tumor activity compared with monotherapy. Combining TCR-transgenic T cells with YB-1-dependent OAV (E1A13S-deleted) effectively targeted Ewing sarcoma xenografts and produced synergistic anti-tumor and immunostimulatory effects.^78^ However, several limitations to ONCOTECH must be addressed before it can be used in clinical practice, including antigenic deficiency and T-cell depletion.

### OVs in combination with TIL therapy

TILs^79^ are a type of immune cell that can kill tumors, with strong tumor targeting, minimal side effects, a large expansion number, and the ability to kill tumors. TIL therapy differs from CAR-T and TCR-T cell therapies in that T cells do not need to be genetically modified. In addition, in cervical cancer,^80^ ovarian cancer,^81^ and especially metastatic melanoma,^82^ TIL therapy has proven to be an effective anti-tumor treatment. In summary, TIL therapy has certain unique advantages in the treatment of tumors, but it still faces a series of challenges such as insufficient activation and low persistence of TILs^83^ and the immunosuppressive TME.^84^ The exploration of combining TIL therapy with OVT is underway, improving the safety of systemic and local administration, and offering new hope for tumor patients.^85^, ^86^, ^87^

In ovarian and pancreatic carcinoma mouse models, a combination of TIL therapy with OVs is being investigated to improve anti-tumor activity.^86^^,^^88^^,^^89^ An engineered OAV, Ad5/3-E2F-d24-vIL2, which encoded a variant IL-2 cytokine, stimulated high granzyme B production, selectively replicated, and destroyed tumor cells to enhance the effectiveness of TILs in ovarian cancer.^89^ What's more, systemic delivery of OAV, which encoded OX40L and IL12 to tumors using TIL as carriers in hamster and mouse models, induced the infiltration of further TILs and enhanced synergistic anti-tumor effects.^90^ A single-arm, multicenter phase I dose escalation trial ^91^ enrolled 20 patients with sarcomas, melanomas, and ovarian cancers to evaluate the safety of an OAV (TILT-123), which encoded TNF-α and IL-2 in combination with TIL therapy. Results of the study showed that the combination therapy with OAV and TIL was safe and effective in the treatment of patients with metastatic melanoma. Combination therapy produced anti-tumor effects on patients with local and distant lesions and had broad clinical application prospects. However, more preclinical studies are required to validate its efficacy, clinical effectiveness, and safety.

### OVs in combination with immune checkpoint therapy

Immune checkpoints are immunosuppressive molecules expressed on immune cells, which regulate the degree of immune activation, preventing the occurrence of autoimmune effects.^92^ The goal of immune checkpoint therapy is to block the inhibitory signal of T cell activation to boost the immune response against tumors.^93^ There are currently a variety of immune checkpoint inhibitors (ICIs) approved by the National Medical Products Administration of China, including programmed death-1 (PD-1) inhibitors, programmed death ligand-1 (PD-L1) inhibitors, cytotoxic T-lymphocyte-associated protein 4 (CTLA-4) inhibitor, and PD-1/CTLA-4 inhibitor.^94^^,^^95^ Although immune checkpoint therapy produced persistent immune responses in terminally tumor patients, only a small percentage of patients benefited^96^ and relapse often occurred due to multiple resistance mechanisms as well as the complex TME.^97^ OVs in combination with ICIs could not only improve the presentation of DC cells and the activation of T lymphocytes but also enhance the function of effector T cells and improve anti-tumor efficacy.^98^^,^^99^

### OVs in combination with PD-1/PD-L1 inhibitors

PD-1 is a member of the CD28 superfamily, the main immune checkpoint found on T cells, and can be expressed on the surface of immune cells.^100^ T-cell signal transduction is interfered with by the interaction between PD-1 and PD-L1.^101^ By developing antibodies that can block the PD-1/PD-L1 axis, great success has been achieved in the immunotherapy of malignant tumors. Even though PD-1/PD-L1 inhibitors have been known as “anti-tumor miracle drugs”, their application prospects are limited by immune-related adverse events, such as diarrhea, colitis, infections, and infestations.^102^ There may be a safer and more effective combination of OVs with PD-1/PD-L1 inhibitors when used simultaneously.^103^, ^104^, ^105^, ^106^, ^107^

A phase Ib trial (NCT04197882) evaluated the efficacy and safety of combining OV orienX010, which encoded GM-CSF, with the PD-1 inhibitor toripalimab for the treatment of acral melanoma. The combination significantly increased cytokine and chemokine secretion and showed encouraging anti-tumor effects and high response rates.^108^ In addition, a multicenter phase 1/2 study (NCT02798406) evaluated the combination of OV-DNX-2401 with the anti-PD-1 antibody pembrolizumab in recurrent glioblastoma multiforme.^109^ OV-DNX-2401 was characterized by two stable genetic modifications in the adenovirus dsDNA genome, which enabled selective and efficient replication. The median overall survival was 12.5 months, and 56.2 % of patients had stable or improved disease. Overall, DNX-2401 in combination with pembrolizumab was safe and effective and showed significant survival benefits. The combination of OVs with PD-1/PD-L1 inhibitors has been found to be an effective anti-tumor strategy that can reshape the TME and induce anti-tumor T cell immunity.^106^^,^^110^ However, PD-1/PD-L1 is most effective primarily in primary tumors, so it is difficult to ensure systemic effects from monotherapy.^111^ The combination therapy presents^112^ a new method to improve the effectiveness of OVs and overcome tumor immune tolerance, thus meanwhile providing a strong foundation for the use of recombinant OV medications expressing PD-1 single-chain antibodies in clinical tumor therapy.^6^

### OVs in combination with CTLA-4 inhibitors

CTLA-4 is expressed on the surface of activated T cells and is homologous to CD28, both of which bind to CD80 and CD86 on the surface of antigen-presenting cells.^113^ On the one hand, it mediates the immunosuppressive ability of regulatory T cells; on the other hand, CTLA-4 is expressed after activation of resting T cells.^114^ As CTLA-4 plays an important role in driving regulatory T cells, blocking it increases the intra-tumoral immune response by attenuating tumor-infiltrating regulatory T cells.^115^ However, the treatment of single CTLA-4 inhibitors has shortcomings and challenges in tumor heterogeneity, tumor drug resistance, and tumor recurrence.^116^ Moreover, recent studies have indicated that the combination of OVs with CTLA-4 inhibitors offers a promising approach for the treatment of tumors.^117^, ^118^, ^119^

OncoVEXm^GM−CSF^ was an OV that encoded mouse GM-CSF and changed the TME to enhance responsiveness to anti-CTLA-4 antibody therapy. The combination increased immune infiltrate and necrosis in distant HSV-1-refractory lung metastases, ultimately leading to the establishment of durable immune memory and prolonged animal survival in mouse models of melanoma.^120^ Another randomized phase II study (NCT01740297) enrolled 198 patients with advanced melanoma who received ipilimumab (*n* = 100) or a combination of talimogene laherparepvec (T-VEC) plus ipilimumab therapy (*n* = 98).^121^ Objective response rate was 35.7% in the combination arm and 16.0% in the ipilimumab arm. Combination therapy showed higher anti-tumor activity overall, without additional toxicity. TG6050, an oncolytic vaccinia virus (OVV) encoding IL-12 and anti-CTLA-4 (@CTLA-4), allowed prolonged intra-tumoral expression of IL-12 and @CTLA-4 at effective levels without systemic toxicity and promoted tumor regression through significant immune remodeling of the TME.^122^ Although the combination of OVs with CTLA-4 inhibitors offers a glimmer of hope for tumor patients, most studies are still in their infancy.^123^ There is a need for a more rational design of drug injection routes, sequences, and doses of combination therapy.

### OVs in combination with other ICIs

Furthermore, apart from the aforementioned PD-1/PD-L1, CTLA-4 inhibitors, numerous other target inhibitors are currently being developed, including lymphocyte activation gene-3 (LAG-3),^124^ T cell immunoglobulin domain and mucin domain-3 (TIM-3),^125^ T cell immunoreceptor with Ig and ITIM domain (TIGIT),^126^ V-domain Ig suppressor of T-cell activation (VISTA), and B and T lymphocyte attenuator (BTLA)^127^ inhibitors. Because the therapeutic potential of OVs is influenced by the tumor immunosuppressive TME, it is necessary to arm OVs with ICIs. In a recent study, a new recombinant OV, VV-α-TIGIT, was designed.^128^ It combines the advantages of OV and ICI successfully. *In vivo* and *in vitro* experiments showed that VV-α-TIGIT significantly increased the recruitment and activation of T cells in the TME, leading to improved anti-tumor efficacy. Zuo et al^129^ armed an OVV with a single-chain variable fragment (scFv) of TIGIT to investigate the anti-tumor effect in multiple animal models. In addition, VV-scFv-TIGIT in combination with PD-1 inhibition or LAG-3 inhibitors was investigated for anti-tumor efficacy in colon tumor models. Results of an *ex vitro* study showed that the combination therapy of an OVV, along with immune checkpoint blockade, led to a higher anti-tumor activity than monotherapy. A previous study showed that an OV expressing hPD-1scFv could safely and effectively enhance systemic anti-tumor immunity by boosting T cells and overcoming local immunosuppression to make tumors more responsive to CTLA-4 or TIM-3 blockade.^130^ These results suggest that OVs can be used as a safe and effective auxiliary tool to cooperate with ICIs to achieve anti-tumor effects through multiple mechanisms. In addition, more clinical trials of combination therapies are steadily progressing.^130^, ^131^, ^132^ A new generation of immune checkpoints is emerging in an endless stream, and their signal transduction mechanisms and ligands' functions are still unclear, so a large number of experiments are still needed to further study their mechanisms to design a more reasonable combination therapy with OVs.

### OVs in combination with cancer vaccines

Tumor antigens are commonly categorized into two groups, tumor-specific antigens and tumor-associated antigens. The rationale for the development of a cancer vaccine is to enhance the immune system's ability to recognize and kill tumor cells containing specific antigens by targeting tumor-specific antigens or tumor-associated antigens.^133^ Cancer vaccines can include DNA vaccine, mRNA vaccine, peptide vaccine, and DC vaccine,^134^^,^^135^ and have been used in clinical trials to treat patients with multiple solid tumors, such as prostate cancer, non-small cell lung cancer, melanoma, colon carcinoma, ovarian cancer, advanced hepatocellular carcinoma, and glioma.^136^, ^137^, ^138^, ^139^ However, cancer vaccines remain hindered by immunosuppressive TME and tumor-associated antigen deficiency.^140^ As OVT can overcome immunosuppression and expose tumor-associated antigens, it may strengthen the anti-tumor efficacy of cancer vaccine combination therapy.

For example, OV M1 (OVM) mitigated DC inhibition and restored its function of priming T cell response by down-regulating signal regulatory protein alpha (SIRRPα) on the surface of DCs and CD47 on the surface of tumor cells.^140^ Compared with monotherapy, combining the two treatments resulted in significantly delayed tumor growth and increased survival time, suggesting that OVM could enhance DC vaccine's anti-tumor efficacy in syngeneic tumor mouse models.^140^ Similar preclinical therapeutic results were also observed when melanoma-targeting vaccines were combined with GM-CSF armed vesicular stomatitis virus (VSV).^141^ In addition, OAV, which was enriched with CpG motifs, combined with the poly-epitope pDNA vaccine improved the anti-tumor activity of the pDNA vaccine, stimulated a more robust antigen-specific immune response, and enhanced tumor regression in mouse models of melanoma.^142^ In tumor treatment, novel cancer vaccines, particularly those based on individual-specific neoantigens, represent a new direction. For example, mRNA vaccines^143^ are used to develop personalized anti-tumor vaccinations that can effectively activate the immune system and improve the ability to recognize and kill tumor cells through accurate selection of antigen targets. In preclinical research, Fu et al generated an OV (rVSV-LCMVG) by substituting the VSV glycoprotein with the LCMV glycoprotein gene, which was codon-optimized for expression in human cells.^144^ The rVSV-LCMVG enhanced the anti-tumor efficacy of the tumor-targeting mRNA vaccines, improved the response to melanoma, and prolonged the survival of mice.^144^ Due to its instability, difficulty in preservation, and ineffective delivery, RNA poses a great challenge. Trials will continue to be conducted to develop the most effective preservation vector for mRNA vaccines,^145^ simplify the delivery method to improve targeting and push immunotherapy and tumor therapy into a new era.

### OVs in combination with BiTEs or TriTEs

BiTEs, a new type of immunotherapeutic agents, are fusion proteins consisting of two antibody scFvs with dual antigen-binding sites,^146^ one of which binds specifically to tumor-associated antigens on tumor cell surfaces, and the other binds to CD3 or other T cell activators. This enables the effective activation of effector T cells, leading to the elimination of tumor cells. TriTEs possess three distinct antigen-binding sites, which allow them to interact with both target cells and functional cells (typically T cells or NK cells).^147^ Although BiTEs or TriTEs can improve the accuracy and efficacy of current immunotherapy, their short serum half-life necessitates continuous infusions, and systemic administration is associated with severe side effects.^148^ OVs can address these challenges and enhance the anti-tumor effectiveness of combination therapy with BiTEs or TriTEs.^149^^,^^150^

A recent study developed an HSV-1-based OV armed with Claudin18.2-targeting BiTE to treat pancreatic cancer. The BiTE-armed OV redirected T cells to destroy tumor cells, reshaped the TME, and effectively retarded tumor growth.^151^ Lei et al developed an OVV encoded with a CD19-specific BiTE, referred to as OVV-CD19BiTE. This innovative construct effectively recruited immune cells to the TME and stimulated T-cell proliferation, thereby overcoming the limitations of BiTEs in an immunosuppressive TME. Importantly, the integration of BiTE expression cassettes into OVV did not compromise its replicative or oncolytic capabilities. The OVV-CD19BiTE showed superior anti-tumor efficacy compared with blinatumomab, an anti-CD19 BiTE, in both *in vivo* and *in vitro* experiments.^152^ The study provided strong evidence for the treatment of B-cell lymphoma with OVV-combined BiTEs. Similar therapeutic results were also observed when oHSV-1 G207 was combined with NKG2D BiTE in glioblastoma multiforme.^153^ Furthermore, Khalique et al armed oHSV-1 with PD-L1 BiTE and demonstrated successful facilitation of improved activation of endogenous T cells and enhanced target cytotoxicity toward tumor cells. This study confirmed the anti-tumor efficacy of the treatment across various human cell lines.^154^ What's more, preclinical studies of OVs in combination with TriTEs are ongoing. Taken together, these studies suggest that BiTE-armed OVs are capable of selectively targeting malignant cells as well as tumor stroma, increasing therapeutic efficacy. Therefore, OVs combined with BiTEs or TriTEs have great potential as a tumor treatment in the future.

### OVs in combination with cytokines

Cytokines^155^ are a type of small molecular weight proteins/peptides that can transmit information between cells and have the functions of immune regulation and effect. They can be classified into interferons, tumor necrosis factors, interleukins, colony-stimulating factors, growth factors, and chemokines.^156^ In many immune-mediated diseases, cytokines play an important role. In the past 40 years, cytokines and their receptors have been extensively studied as tumor targets or therapeutic strategies. However, the short half-life, pleiotropy, and adverse tissue distribution of cytokines contribute to their narrow therapeutic range.^157^ The combination of cytokines with OVs has produced a lot of promising results in preclinical studies.

### OVs in combination with GM-CSF

As a common cytokine, GM-CSF enhances antigen presentation during virotherapy by recruiting, activating, and maturing monocytes and DCs.^158^, ^159^, ^160^GM-CSF has been armed to different viruses, including HSV, VV, and AdV, and has been studied in preclinical and clinical trials for various types of tumors.^161^, ^162^, ^163^ For example, ONCOS-102, an OV encoding GM-CSF, enhanced T-cell infiltration and augmented the anti-tumor efficacy in multiple tumors.^164^ Shi et al developed a modified Sindbis virus, SINV-GM-CSF, which carried recombinant GM-CSF. The inclusion of GM-CSF improved the virus's tumor cell killing and boosted immune cell presence in the TME.^165^ In a mouse model of hepatocellular carcinoma, SINV-GM-CSF showed significant tumoricidal activity by modulating the increase of M1-type macrophages and the reduction of M2-type macrophages.^165^ Similar preclinical therapeutic results were also observed when GM-CSF and IL-12 oHSVs were combined with α-PD1.^166^ In summary, these studies demonstrate the great potential of OVs in combination with GM-CSF in the treatment of tumors.

### OVs in combination with IL-2

IL-2 was the first factor to receive clinical approval for tumor therapy, and high-dose IL-2 is approved for treating advanced renal cell carcinoma and melanoma. As a result of its short half-life and low tumor accumulation, systemic IL-2 therapy does not deliver the anticipated anti-tumor effects.^167^ In recent studies, researchers successfully engineered OV coding IL-2 to facilitate localized expression of IL-2 within tumors, thereby augmenting the anti-tumor efficacy of OVs, which was validated in *in vivo* and *in vitro* experiments.^168^, ^169^, ^170^ For example, Bommareddy et al developed G47Δ-mIL2 (an oHSV expressing IL-2) to deliver IL-2 locally within the TME.^171^ This treatment regimen avoided the systemic IL-2-related therapeutic deficiencies while generating beneficial anti-tumor immunity in glioblastoma multiforme. This indicates that the G47Δ-mIL2 virus, which expressed both mCherry and murine IL-2, does not hinder viral entry, replication, or cytotoxicity.

### OVs in combination with IL-21

IL-21 is primarily secreted by activated CD4 T cells and plays a pleiotropic role in the regulation of many types of immune cells, including B cells, T cells, NK cells, and DC cells.^172^ Recent research indicates that IL-21 is capable of suppressing tumor growth. However, increasing evidence suggests that monotherapy usually is not adequate to stop tumor progression. Combination therapies are usually more effective. Chen et al engineered a recombinant thymidine kinase-deleted TTV752-1 (rTTVΔTK) as a backbone to generate a mouse IL-21 (mIL21)-armed recombinant TTV (rTTV). IL-21 arming augmented the function of CD4^+^ and CD8^+^ T cells, potentiated the anti-tumor activity of OVV, and reshaped the tumor immune microenvironment in mouse models of glioblastoma multiforme.^173^ Another study also investigated an effective therapeutic regimen for the treatment of glioma using OVV expressing mIL-21 (VVΔTK-STCΔN1L-mIL-21) in combination with immune checkpoint inhibition.^174^ VVΔTK-STCΔN1L-mIL-21 effectively activated splenic T cells eliminated GL261 subcutaneous tumors in mice, and achieved lasting tumor rejection and better survival rates.^174^ In summary, the results of studies demonstrate the great potential of OVs in combination with IL-21 in the treatment of tumors.

### OVs in combination with other cytokines

In addition to the above-mentioned cytokines, other cytokines including IL-23, IL-36, IL-15^175^^,^^176^, IL-18^177^^,^^178^, and IL-7^179^^,^^180^ are candidate cytokines to arm OVs for tumor immunotherapy. For example, by homologous recombination, OVVs expressing the IL-23 variant were designed, which elicit powerful anti-tumor effects on various tumor models by regulating TME.^181^ IL-23 extended viral persistence through the up-regulation of IL-10, while its sustained expression and viral oncolysis enhanced chemokines and anti-tumor factors, making the TME more favorable for anti-tumor immunity.^181^ Similarly, Yang et al designed an oncolytic VV expressing the active form of IL-36γ based on the direct oncolytic features and tumor selectivity of VV,^182^ as well as the immunostimulatory effects of IL-36γ. IL-36γ-armed OVs promoted anti-tumor efficacy, reshaped the TME, and showed significant therapeutic efficacies in multiple mouse tumor models. The combination of OVs with cytokines has promise as an anti-tumor agent, so it merits further clinical exploration.

## Future perspectives and conclusions

In recent years, the advancement of genetic engineering technology and the enhanced comprehension of virus structure and function have propelled oncolytic virotherapy to a significant breakthrough in tumor treatment, establishing it as a crucial modality for tumor management. Currently, OVs are primarily utilized in the treatment of solid tumors, such as melanoma, hepatocellular carcinoma, colorectal cancer, breast cancer, glioblastoma multiforme, head and neck cancers, and glioma.^7^ Among them, some tumors exhibit heightened sensitivity to OVs, such as melanoma and glioma. These tumors typically display a lower immunosuppressive state, which enables OVs to more effectively infect and destroy tumor cells while triggering the host's anti-tumor immune response. Currently, four OV products have been approved worldwide for the treatment of melanoma,^24^^,^^183^ glioma,^184^ and nasopharyngeal cancer,^185^ and OVs have been demonstrated to have significant anti-tumor efficacy in these malignancies. It is imperative to not only intensify the comprehensive investigation of these specific tumors but also to extend research efforts to other tumor types to facilitate broader clinical applications.

The combination of OVs with ACT, ICIs, cancer vaccines, cytokines, or BiTEs/TriTEs displays significant potential in effectively managing tumor progression, thereby presenting a novel clinical therapeutic avenue for individuals afflicted with tumors. Although numerous clinical trials have consistently validated the favorable effectiveness and durability of OV-combined immunotherapy, there are still some limitations to be solved urgently. First, successful delivery of OVs to the tumor site with high concentration and vitality is the primary challenge for OVT. Intra-tumoral injections are the best method for accomplishing this. However, intra-tumoral injections are limited by their applicability and they require a high level of technical skill. Fortunately, this problem has been partly solved due to the emergence of multiple novel delivery tools, including immune cells, stem cells, nanoparticles, and hydrogel.^186^^,^^187^ Second, there is a risk of increased toxicity associated with combination therapies, such as cardiotoxicity after treatment.^188^ However, this has been challenging so far, and further mechanistic studies are needed to fully understand how various treatment combinations mediate anti-tumor activity, thereby improving side effects. Finally, tumor treatment must be based on the full understanding of individual differences, taking an individualized comprehensive treatment plan. Therefore, the appropriate combination strategy and OV type should be selected according to the specific situation of each patient.

Gaining a comprehensive understanding of the immunobiology of OV therapy will significantly advance the development of advanced viral strategies and offer the potential for curative responses among patients. Therefore, in-depth basic research, preclinical research, and large-scale clinical trials are still needed to explore more anti-tumor mechanisms of combination therapies, improve their safety and efficacy, and establish new diagnosis and treatment programs to comprehensively improve the clinical benefit and quality of life of tumor patients. Combination therapy of OVs with tumor immunotherapies is expected to yield better results in the future.

## CRediT authorship contribution statement

**Xiaoli Zhou:** Writing – original draft. **Shunfeng Hu:** Writing – review & editing. **Xin Wang:** Writing – review & editing.

## Funding

This study was funded by the 10.13039/501100001809National Natural Science Foundation of China (No. 82270200, 82070203, 81770210, 82400231); Taishan Scholars Program of Shandong Province, China; Shandong Provincial 10.13039/100000084Engineering Research Center of Lymphoma (China); 10.13039/100014103Key Research and Development Program of Shandong Province, China (No. 2018CXGC1213); Academic Promotion Programme of 10.13039/501100015507Shandong First Medical University (China) (No. 2019QL018); Translational Research Grant of the National Clinical Research Center for Hematologic Diseases (NCRCH) (China) (No. 2021WWB02, 2020ZKMB01); 10.13039/501100002858China Postdoctoral Science Foundation (No. 2023M741506); and 10.13039/501100007129Shandong Provincial Natural Science Foundation (China) (No. ZR2023QH193).

## Conflict of interests

The authors declared no conflict of interests.
